# Vesicular stomatitis virus replicon expressing the VP2 outer capsid protein of bluetongue virus serotype 8 induces complete protection of sheep against challenge infection

**DOI:** 10.1186/1297-9716-45-64

**Published:** 2014-06-13

**Authors:** Stefanie Kochinger, Nathalie Renevey, Martin A Hofmann, Gert Zimmer

**Affiliations:** 1 Institute of Virology and Immunology (IVI), Sensemattstrasse 293, CH-3147 Mittelhäusern, Switzerland

## Abstract

Bluetongue virus (BTV) is an arthropod-borne pathogen that causes an often fatal, hemorrhagic disease in ruminants. Different BTV serotypes occur throughout many temperate and tropical regions of the world. In 2006, BTV serotype 8 (BTV-8) emerged in Central and Northern Europe for the first time. Although this outbreak was eventually controlled using inactivated virus vaccines, the epidemic caused significant economic losses not only from the disease in livestock but also from trade restrictions. To date, BTV vaccines that allow simple serological discrimination of infected and vaccinated animals (DIVA) have not been approved for use in livestock. In this study, we generated recombinant RNA replicon particles based on single-cycle vesicular stomatitis virus (VSV) vectors. Immunization of sheep with infectious VSV replicon particles expressing the outer capsid VP2 protein of BTV-8 resulted in induction of BTV-8 serotype-specific neutralizing antibodies. After challenge with a virulent BTV-8 strain, the vaccinated animals neither developed signs of disease nor showed viremia. In contrast, immunization of sheep with recombinant VP5 - the second outer capsid protein of BTV - did not confer protection. Discrimination of infected from vaccinated animals was readily achieved using an ELISA for detection of antibodies against the VP7 antigen. These data indicate that VSV replicon particles potentially represent a safe and efficacious vaccine platform with which to control future outbreaks by BTV-8 or other serotypes, especially in previously non-endemic regions where discrimination between vaccinated and infected animals is crucial.

## Introduction

Bluetongue is a hemorrhagic disease of ruminants that is caused by bluetongue virus (BTV), a member of the genus Orbivirus within the family *Reoviridae*[1-3]. BTV is transmitted to livestock by blood-feeding *Culicoides* midges. In cattle, goats, and wild ruminants, BTV infection is typically asymptomatic despite prolonged viremia. These host species represent a potential reservoir for unnoticed dissemination of BTV in ruminant populations. In sheep, however, BTV infection often results in an acute disease with associated high morbidity and mortality, depending on the virulence of the virus and the sheep breed affected [4]. Typical symptoms of bluetongue in sheep include high fever, tissue edema, hemorrhages, and necrosis of the upper gastrointestinal tract as well as of skeletal and cardiac musculature. Certain strains of BTV, notably the northern European strain of BTV-8, can cross the placental barrier, leading to infection of the developing fetus [5]. Hence, infection of pregnant animals with certain strains of the virus are frequently associated with abortions and malformations of offspring [6-8].

The BTV genome consists of 10 segments of dsRNA, which encode 7 structural (VP1 - VP7) and 5 non-structural proteins (NS1 – NS4, NS3a) [9]. The non-enveloped icosahedral virion particle contains an inner core which is formed by the viral RNA and the viral proteins VP1 (RNA polymerase), VP4 (capping enzyme), and VP6 (helicase) [10,11]. The inner core is surrounded by 3 concentric protein layers. The inner capsid layer consists of 120 copies of VP3 arranged as 60 dimers that form a scaffold for VP7. The outer capsid is composed of the viral proteins VP2 and VP5, which are responsible for receptor binding, hemagglutination, and membrane penetration, respectively [12,13]. The large (110 kDa) attachment protein VP2 induces virus-neutralizing antibodies [14]. However, VP2 is highly variable and currently 26 different BTV serotypes can be distinguished by antibodies that show little or no cross-neutralizing activity [3]. Binding of VP5 to VP2 leads to a VP2 conformational change, which may affect recognition of epitopes by neutralizing antibodies [15,16]. All other structural and non-structural proteins are relatively conserved among different BTV serotypes. Therefore, most ELISAs for pan-BTV antibody detection employ the VP7 antigen [17].

A novel strain of BTV serotype 8 (BTV-8), which had not been detected in Europe before 2006, emerged as an epidemic wave in Western and Northern Europe [3,18,19]. This outbreak had a significant economic impact, not only because the disease caused morbidity and mortality in sheep and cattle but also because of restrictions imposed on livestock movement and trade [20]. The epizootic was controlled by a large-scale vaccination campaign using whole inactivated BTV-8. Although this vaccine induced strong protection against BTV-8 infection and disease, it did not allow the simple serological discrimination of infected from vaccinated animals (DIVA). Furthermore, vaccine production required the production of large amounts of infectious virus in cell culture, proper virus inactivation, and formulation of the antigen with adjuvant, all of which delayed production and added to the costs of producing the vaccine. Importantly, inactivated virus vaccines may not be suitable for the control of all serotypes of BTV as, for example, serotype 25 cannot be propagated in cell culture [21]. To overcome these hurdles, our goal was to develop and evaluate a generic vector vaccine in sheep, one that would comply with the DIVA principle. Although similar strategies using recombinant poxviruses (vaccinia, goatpox, and canarypox viruses), herpesviruses, and virus-like particles have been proven effective, we elected to evaluate the vesicular stomatitis virus (VSV) replicon system [22].

VSV is a non-segmented negative-strand RNA virus that is known to trigger a robust humoral immune response in many different host species [23]. Recombinant VSV vectors have been employed for experimental vaccination of mammals against a number of different pathogens such as human papilloma virus, hepatitis C virus, or influenza A virus [24]. A modified VSV vector in which the VSV glycoprotein (G) gene was replaced by the influenza A virus HA gene was shown to protect chickens from challenge infection with highly pathogenic avian influenza viruses [25,26]. This vector was propagated to high titers on helper cells providing the VSV G protein *in trans* and was able to infect a broad spectrum of different cell types. Due to the autonomous replication of the viral RNA, high-level expression of recombinant antigens was achieved. However, due to the deletion of the VSV G protein gene, VSVΔG vectors are restricted to a single round of infection, contributing to their excellent biosafety profile. These novel vector vaccines were effective even though adjuvants were not employed [25,26].

In this study, propagation-incompetent VSVΔG vectors expressing the BTV-8 outer capsid proteins VP2 or VP5, or a combination of both, were generated. The expression of recombinant BTV antigens was studied and the immune response in sheep assessed. Immunized sheep were then infected with virulent BTV-8 and monitored for viremia, clinical symptoms, and antibody production. The DIVA principle was assessed using a commercially available VP7-based competitive ELISA.

## Materials and methods

### Cells

BHK-21 cells were obtained from the German Cell Culture Collection (DSZM, Braunschweig, Germany) and grown in Earle’s minimal essential medium (MEM; Life Technologies, Carlsbad, CA, USA) supplemented with 5% fetal bovine serum (FBS; Biowest Nuaillé, France). BHK-G43, a transgenic BHK-21 cell clone expressing the VSV G protein in a regulated manner, was maintained as described previously [27]. Vero cells (C1008) were purchased from the American Type Culture Collection (Manassas, VA, USA) and maintained in Glasgow’s minimal essential medium (GMEM; Life Technologies) supplemented with 5% FBS.

### Viruses

A German BTV-8 isolate (from 2008) was kindly provided by Bernd Hoffmann (FLI, Riems, Germany). A French isolate of BTV-1 was obtained from The Pirbright Institute (Pirbright, UK). Virus stocks were titrated on Vero cells. Virus infection was monitored taking advantage of the cytopathic effect apparent at day 5 post infection (pi). Infectious virus titres were calculated according to the Spearman-Kärber method and expressed as 50% tissue culture infectious doses per mL (TCID_50_/mL).

In order to obtain virulent BTV-8 for challenge experiments, two adult BTV-seronegative Poll Dorset sheep were inoculated intravenously using erythrocytes prepared from a BTV-8 viremic calf. This virus was originally isolated in 2008 in Gummersbach, Germany. Following infection, blood was collected at daily intervals and analyzed for the presence of viral RNA by RT-qPCR [28]. The sheep were euthanized at day 5 pi, when a quantification cycle (C_q_) value of 18 was determined in blood by RT-qPCR. Blood was collected from the animal, erythrocytes were washed and suspended in PBS, and frozen at −70 °C.

### Construction of recombinant VRPs

Codon-optimized cDNA of VP2 (GenBank accession number AM498052) and VP5 (GenBank accession number AM498056) of the BTV-8 strain NET2006/04 was synthesized by GenScript (Piscataway, NJ, USA). For generation of recombinant VSV, the BTV-8 genes were inserted into the plasmid pVSV* using *MluI* and *BstEII* restriction sites upstream and downstream of the fourth transcription unit, thereby replacing the VSV G gene [25]. The resulting plasmids were designated pVSV*∆G(VP2) and pVSV*∆G(VP5), respectively. For generation of a dual antigen expression vector, the VP5 cDNA was inserted into pVSV*∆G(VP2) using *XhoI* and *NheI* restriction sites located upstream and downstream of the fifth transcription unit, thereby replacing the green fluorescent protein (GFP) gene. The resulting plasmid was designated pVSV∆G(VP2,VP5). For expression of a modified VP5 containing a short peptide epitope at the C terminus, the VP5 gene without its Stop codon was inserted into the pCMV-3Tag-3 plasmid vector (Agilent Technologies, Inc., Santa Clara, CA, USA) upstream of and in frame with a triple FLAG epitope (DYKDDDDK)-coding region, which was followed by a Stop codon. The VP5-FLAG open reading frame was then amplified by PCR and inserted into either the fourth transcription unit of pVSV*∆G or the fifth transcription unit of pVSV*∆G(VP2). The resulting plasmids were designated pVSV*∆G(VP5-FLAG) and pVSV∆G(VP2,VP5-FLAG), respectively. VSV replicon particles (VRPs) were generated and propagated on BHK-G43 helper cells as described previously [29].

Recombinant VSV*∆G(VP2), VSV*∆G(VP5), VSV*∆G(VP5-FLAG), and VSV*∆G [25] were titrated on BHK-21 cells taking advantage of the GFP reporter protein. Infectious titers were calculated and expressed as fluorescence-forming units per milliliter (ffu/mL). For detection of VSV∆G(VP2,VP5) and VSV*∆G(VP2,VP5-Flag), infected cells were fixed with PBS containing 3% paraformaldehyde for 20 min at room temperature, washed with PBS containing 0.1 M (w/v) glycine, and permeabilized with 0.25% (v/v) of Triton-X100. The cells were incubated with rabbit anti-VSV serum and subsequently with a goat anti-rabbit horseradish peroxidase conjugate (dilution 1:500 in PBS; DAKO; Glostrup, Denmark) and finally stained with AEC/H_2_O_2_ substrate.

### Immunofluorescence analysis

Vero cells grown on 12-mm-diameter cover slips were inoculated for 90 min with either VSV*ΔG, VSV*ΔG(VP2), VSV*ΔG(VP5-FLAG), VSVΔG(VP2,VP5) or pVSV∆G(VP2,VP5-FLAG) using a multiplicity of infection (MOI) of 3. At 8 hours pi, the cells were fixed with 3% paraformaldehyde for 20 min, washed with PBS containing 0.1 M (w/v) glycine, and incubated with PBS containing 0.25% (v/v) Triton X-100 for 5 min to permeabilize the plasma membrane. The cells were incubated with a monoclonal antibody directed against either VP2 (clone 13C10, diluted 1:10; kindly provided by Dr Malte Dauber, FLI Riems, Germany) or a monoclonal antibody directed against the FLAG epitope (clone M2, diluted 1:50; Sigma-Aldrich, Deisenhofen, Germany) and subsequently with an anti-mouse IgG-Alexa 546 conjugate (dilution 1:500; Life Technologies, Carlsbad, CA, USA). Finally, the cells were washed with distilled water and embedded in Mowiol 4–88 (Sigma-Aldrich, Deisenhofen, Germany) mounting medium. The cells were analyzed using a Leica TCS-SL spectral confocal microscope (CFM) and Leica LCS software (Leica Microsystems AG, Glattbrugg, Switzerland).

To analyze sera from immunized sheep for the presence of virus-specific antibodies, Vero cells were grown on 12-mm-diameter cover slips, infected with BTV-8 (MOI of 1), and incubated for 48 h with medium containing 0.9% (w/v) methylcellulose. The cells were fixed, permeabilized, and incubated with serum (diluted 1:100 in PBS with 1% BSA) from immunized sheep and subsequently with anti-sheep IgG-Alexa 488 conjugate (dilution 1:500 in PBS; Life Technologies, Carlsbad, CA, USA). Fluorescence microscopy was performed using an Axio Observer Z1 inverted microscope (Zeiss, Jena, Germany).

### Animal experiments

Animal trials were performed in compliance with the Swiss Animal Protection Law and approved by the Animal Welfare Committee of the Canton of Berne (authorization number 112/12). Twenty-four white Swiss White Alp sheep (12 to 24 months old), that were tested seronegative for BTV by ELISA (see below), were immunized intramuscularly with 2.5 mL of cell culture supernatant containing either VSV*ΔG, VSV*ΔG(VP2), VSV*ΔG(VP5), or VSVΔG(VP2,VP5), respectively. Three weeks (day 21) after the primary immunization (day 0), the animals received the same vaccine a second time. At day 42, 4 mL of erythrocyte suspension from BTV-8 infected viremic sheep (see above) were injected into the animals intravenously. Following inoculation, the animals were surveyed daily for clinical signs of disease which were scored according to a modified clinical scoring system [30]. All surviving animals were euthanized at day 14 post challenge.

### Serological tests

Serum neutralization tests were performed as described previously [31]. Briefly, immune sera (heat-inactivated at 56 °C for 30 min) were serially diluted in FBS-free tissue culture medium and incubated for 1 h at 37 °C with 40 TCID_50_/50 μL. Vero cells were added and incubated at 37 °C for 5 days. If not neutralized by immune serum, BTV caused a cytopathic effect 3 to 5 days pi. Neutralizing antibody titres were calculated according to the Spearman-Kärber method. For detection of VSV-neutralizing antibodies, a recombinant VSV (serotype Indiana) expressing GFP was used [32]. A polyclonal anti-VSV serum was used as reference. For detection of VP7-specific serum antibodies, a commercially available competitive ELISA was used according to the manufacturer’s protocol (VMRD, Pullman, USA).

### RNA extraction and quantitative RT-PCR

Total RNA was extracted from whole blood samples using the Ambion MagMAX blood RNA isolation kit (Applied Biosystems, Foster City, California, USA) and stored at −70 °C. For detection of viral RNA, a reverse transcriptase quantitative PCR (RT-qPCR) based on the amplification of segment 10 was performed in duplicates using in vitro-transcribed GFP RNA as internal control [28].

### Statistical analysis

Statistical significance (*p* < 0.05) was considered using a two-way ANOVA and multiple comparisons were assessed using GraphPad Prism6 Sidak’s post-hoc test.

## Results

### Generation of recombinant RNA replicon particles expressing BTV antigens

**Figure 1:**
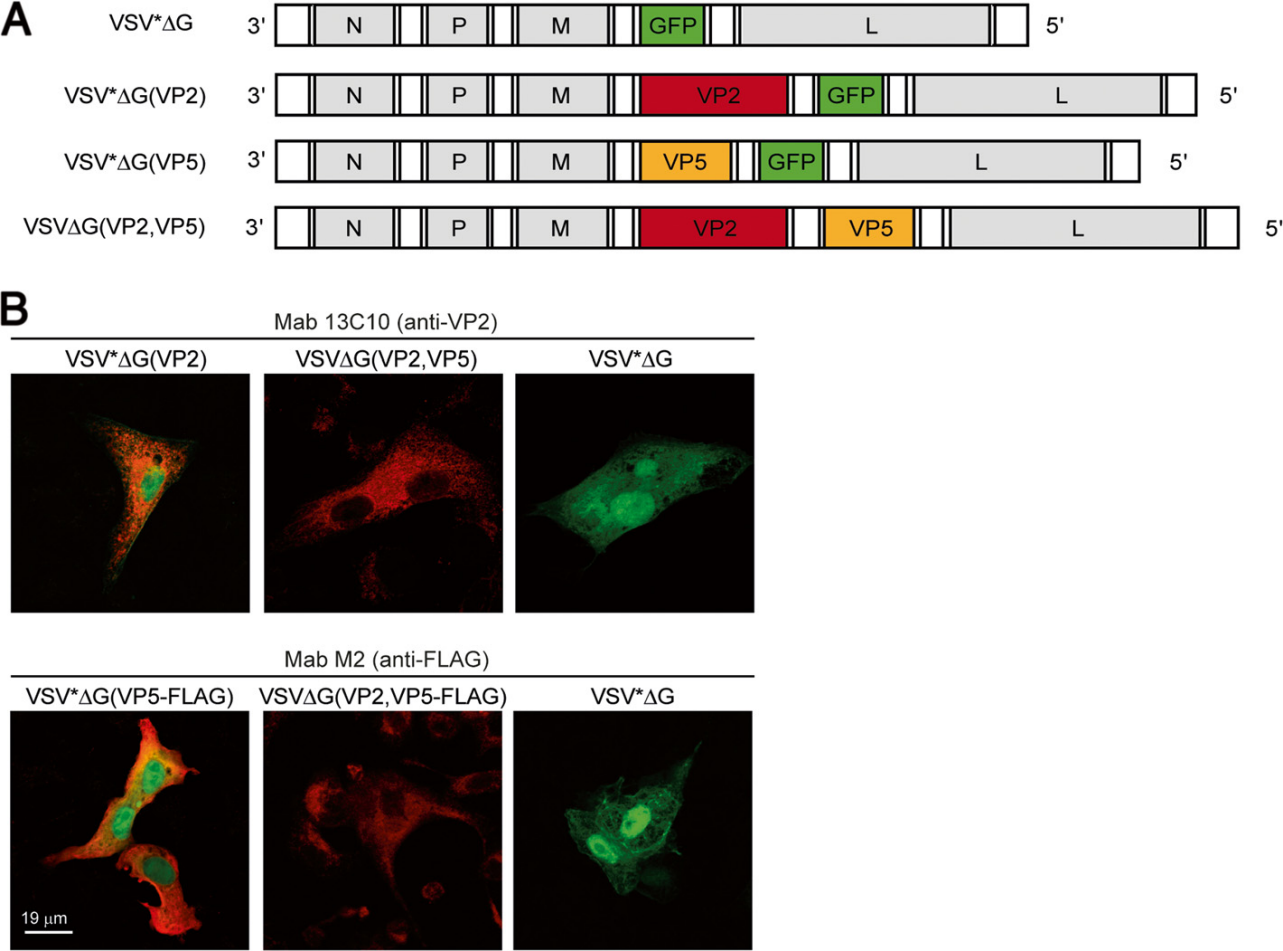
**Genome maps and analysis of recombinant protein expression. (A)** Genome maps of recombinant VSV. VSV*∆G encodes for nucleoprotein (N), phosphoprotein (P), matrix protein (M), and the large RNA polymerase (L). The VSV glycoprotein (G) has been replaced by the GFP gene (denoted by *). VSV*∆G(VP2) and VSV*∆G(VP5) express either VP2 or VP5 antigen of BTV-8 from the fourth transcription unit while GFP is expressed from an additional transcription unit downstream of VP2. VSV∆G(VP2,VP5) expresses VP2 from the fourth and VP5 from the fifth position. **(B)** Immunofluorescence analysis of Vero cells 8 hpi with either VSV*∆G(VP2), VSV∆G(VP2,VP5), VSV*∆G(VP5), VSV*∆G(VP5-FLAG), VSV∆G(VP2,VP5-FLAG), or VSV*∆G. In the upper panel, expression of VP2 is indicated by red fluorescence using a monoclonal anti-VP2 antibody (clone 13C10). In the lower panel, expression of VP5 is indicated by red fluorescence using a monoclonal anti-FLAG (clone M2) antibody. Expression of GFP is indicated by green fluorescence. Scale bar represents 19 μm.

We previously generated a propagation-incompetent VSV vector by replacing the VSV glycoprotein (G) gene in the fourth transcription unit of the viral genome with genes encoding influenza virus antigens [25]. This vector contained an additional transcription unit at position 5, which was used to express the green fluorescent protein (GFP). Based on this vector platform we generated the recombinant viruses VSV*ΔG(VP2) and VSV*ΔG(VP5) containing the VP2 and VP5 genes of BTV-8 in the fourth transcription unit of the vector (Figure 1A). A vector expressing both antigens, VSV*ΔG(VP2,VP5), was constructed by replacing the GFP gene in the fifth position of VSV*ΔG(VP2) by the VP5 gene of BTV-8. VSV*ΔG(VP5-FLAG) and VSVΔG(VP2,VP5-FLAG) contained a triple FLAG epitope at the C terminus of VP5 and were generated to ease detection of this antigen. Recombinant VSV*ΔG served as control vector as it did not express any BTV antigen [25]. All recombinant viruses were propagated on helper cells providing the VSV glycoprotein G *in trans*, which yielded infectious titres of approximately 10^8^ infectious units per mL of cell culture supernatant.The expression of recombinant BTV-8 proteins was determined by immunofluorescence staining of Vero cells 6 hours pi with recombinant VSV. A BTV-8 VP2-specific monoclonal antibody reacted with cells that were infected with VSV*ΔG(VP2) or VSV*ΔG(VP2,VP5), whereas cells infected with VSV*ΔG(VP5) or VSV*ΔG were not recognized (Figure 1B). Since a VP5-specific antibody was not available, expression of VP5 was confirmed using a modified antigen with a triple FLAG epitope at the C terminus. The monoclonal anti-FLAG antibody reacted specifically with Vero cells infected with VSV*ΔG(VP5-FLAG) and VSVΔG(VP2,VP5-FLAG) but not with VSV*ΔG-infected cells (Figure 1B). Taken together, these data show that infection of Vero cells with virus replicon particles readily led to expression of BTV-8 antigens, both VP2 and VP5. However, infectious VSV was not produced by the cells since the VSV G protein was not expressed. Therefore, we refer to the recombinant VSV vectors as virus replicon particles (VRP).

### Analysis of antibody responses in VRP-immunized sheep

**Figure 2:**
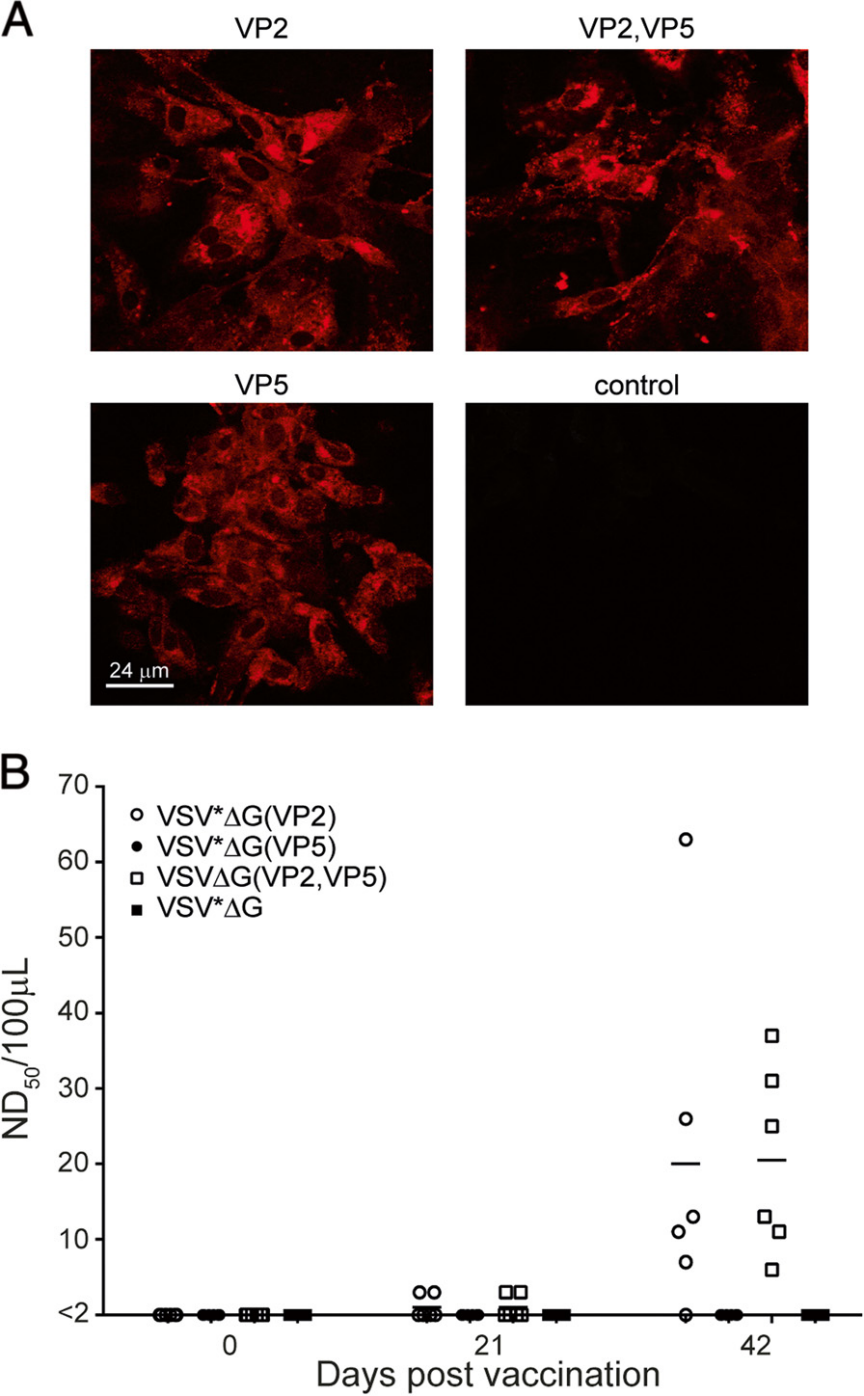
**Antibody responses of immunized sheep. (A)** Immunofluorescence analysis of BTV-8 infected cells. Vero cells were infected with an MOI of 1 and further incubated for 24 h at 37 °C. The cells were fixed, permeabilized, and incubated with immune sera from sheep immunized twice with the indicated VRPs. The primary antibodies were detected with anti-sheep IgG-Alexa 546 conjugate (red fluorescence). Scale bar represents 24 μm. **(B)** Detection of BTV-8 neutralizing serum antibodies. Sera were prepared from VRP-immunized sheep (group size *n* = 6) at day 0 (first vaccination), day 21 (second vaccination), and day 42 (three weeks after the second immunization). Neutralization of virus was estimated 3 to 5 dpi according to the development of CPE. Neutralizing titres were calculated and expressed as ND_50_/100 μL. Values equal to or lower than 2 were regarded negative.

To evaluate the immunogenicity of recombinant VRPs in a natural BTV host, 12 to 24 month-old Swiss White Alp sheep were divided into 4 groups of 6 animals each and immunized with cell culture supernatant containing at least 1 × 10^8^ infectious units of either VSV*ΔG(VP2), VSV*ΔG(VP5), VSVΔG(VP2,VP5), or VSV*ΔG. Adjuvants were not employed. After 3 weeks, the animals were immunized a second time with the same VRPs. No adverse side effects following vaccination were observed. Sera were analyzed 3 weeks after the second immunization for the presence of BTV-specific antibodies by immunofluorescence. Sera from sheep immunized with either VSV*ΔG(VP2), VSV*ΔG(VP5) or VSVΔG(VP2,VP5) reacted specifically with BTV-8 infected cells, indicating that all recombinant antigens were immunogenic and induced the production of BTV-8 specific antibodies (Figure 2A). Sera from VSV*ΔG-immunized sheep did not react.The sheep immune sera were tested for neutralizing activity against BTV-8. Pre-immune sera did not show any neutralizing activity confirming that the animals had no pre-existing immunity to BTV-8 (Figure 2B). Sera collected 3 weeks after the first immunization showed limited neutralizing activity but only at the highest serum concentration used (1:4). However, 3 weeks after the second vaccination with VSV*ΔG(VP2) or VSVΔG(VP2,VP5), all but one of the animals in the VSV*ΔG(VP2) group had developed high neutralizing antibody titres against BTV-8. The same sera did not neutralize BTV-1 or the VSV vector (data not shown). Sheep immunized with either VSV*ΔG or VSV*ΔG(VP5) did not develop BTV-8 neutralizing antibodies. Taken together, these data demonstrate that although recombinant VRPs induced antibodies against both VP2 and VP5, only anti-VP2 antibodies showed BTV-8 neutralizing activity in vitro.

### VRPs expressing VP2 antigen protect sheep from challenge with pathogenic BTV-8

**Figure 3:**
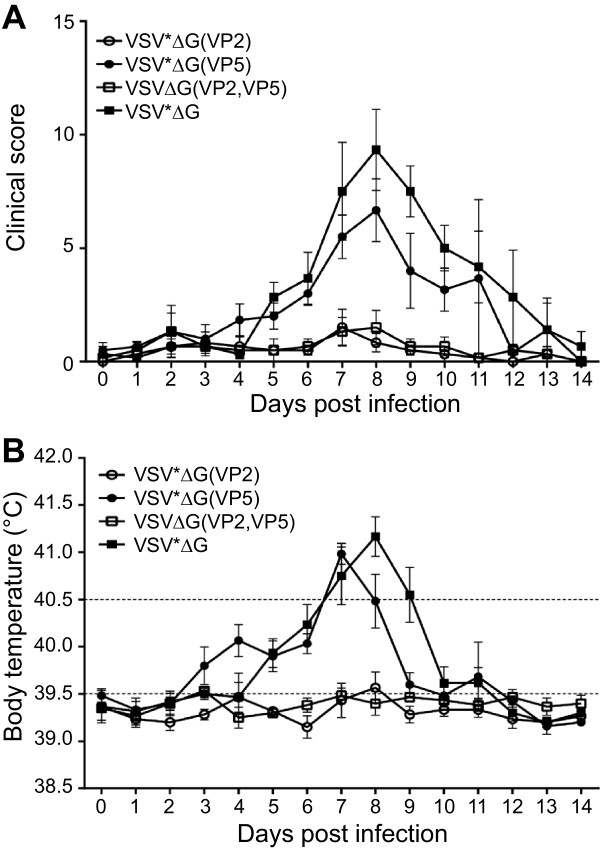
**Clinical signs of disease in BTV-8 infected sheep. (A)** The clinical symptoms of vaccinated sheep groups (*n* = 6) were monitored daily following infection with a virulent BTV-8 strain and scored as described in Materials and Methods. Mean values and standard deviations are indicated. **(B)** The rectal body temperature of the infected animals was determined daily for a total period of 14 days. Body temperatures of up to 39.5 °C were regarded as normal, temperatures between 39.5 °C and 40.5 °C as moderately elevated, and temperatures higher than 40.5 °C were defined as high fever (temperature limits indicated by dashed lines). Mean values and standard deviations are indicated.

**Figure 4:**
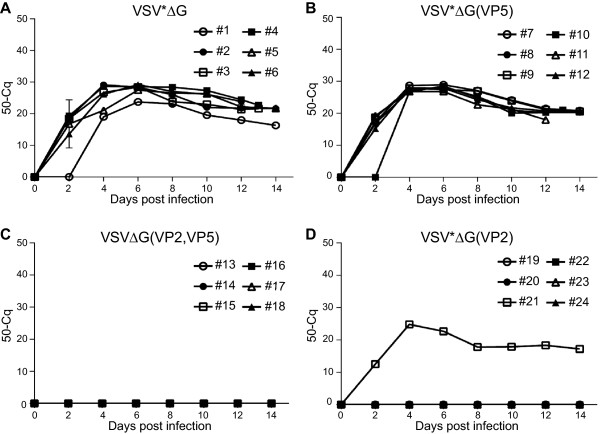
**Determination of viral RNA loads in blood of BTV-8 challenged sheep.** Blood was collected from the animal groups (*n* = 6) vaccinated with **(A)** VSV*ΔG, **(B)** VSV*ΔG(VP5), **(C)** VSV*ΔG(VP2,VP5), and **(D)** VSV*ΔG(VP2) every second day after infection with BTV-8. Total RNA was extracted from whole blood samples and RT-qPCR was performed to determine viral RNA load. The quantification cycles (C_q_) for detection of fluorescence signals were expressed as 50-C_q_ for all 24 animals (numbered #1 to #24).

To evaluate the capacity of recombinant VRP vaccines to induce protective immunity against pathogenic BTV-8, sheep were inoculated intravenously 3 weeks after the second immunization with erythrocyte suspension from a BTV-8 viremic sheep (C_q_ value of 18). Sheep immunized with VSV*ΔG or VSV*ΔG(VP5) showed characteristic signs of bluetongue disease beginning at day 5 pi (Figure 3A). Typical symptoms were facial edema, nasal discharge, and lethargy. Three animals from the VSV*ΔG group and two animals from the VSV*ΔG(VP5) group were euthanized for humane reasons at days 12 and 13 pi, respectively, as they developed severe lung edema and respiratory distress. All animals of the two groups developed high fever, which peaked at day 8 after infection (Figure 3B). In contrast, all animals immunized with VSVΔG(VP2,VP5) and 5 out of 6 animals immunized with VSV*ΔG(VP2) did not show any clinical signs of disease (Figure 3A) and had normal rectal temperature (<39.5 °C) throughout the experiment (Figure 3B). These differences were significant between 7 and 11 dpi in the clinical scoring and between 5 and 9 dpi for body temperature. The single animal in the VP2 group, which did not develop a detectable neutralizing antibody titre to BTV (see Figure 2B), showed moderate clinical signs of disease and fever for three days.The capacity of the vaccine-induced immune response to suppress viremia was evaluated by determining viral RNA loads in blood by quantitative RT-qPCR (Figure 4). Animals immunized with either VSV*ΔG or VSV*ΔG(VP5) had high levels of viral RNA in blood, starting from day 2 post challenge and lasting until the end of the experiment at day 14, whereas BTV RNA was not detected in the blood of the 6 sheep immunized with VSVΔG(VP2,VP5). Likewise, 5 out of 6 sheep immunized with VSV*ΔG(VP2) did not develop any viremia. Thus, recombinant VRPs expressing VP2 alone or in combination with VP5 conferred protective immunity in sheep that prevented both viremia and development of disease.

### VP7 antibodies used for differentiation of infected from vaccinated animals

**Figure 5:**
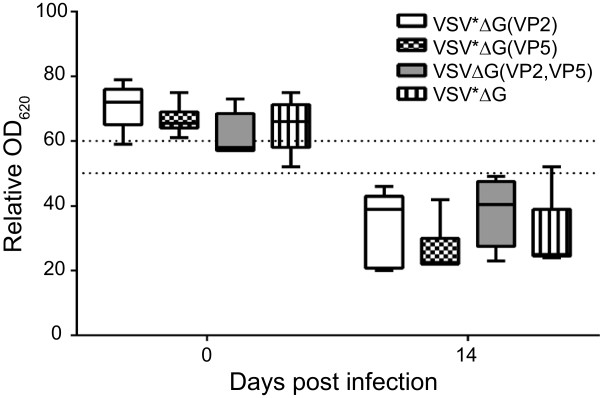
**Detection of VP7 antibodies in serum of immunized sheep before and after challenge infection with BTV-8.** Blood was collected from sheep three weeks after the second immunization (prior to challenge infection with BTV-8) and two weeks after challenge by the end of the experiment. Serum antibodies specific for the VP7 antigen of BTV were detected using a commercially available competitive ELISA. Optical density was recorded at 620 nm and expressed as percentage OD_620_ relative to an internal negative control. OD_620_ values < 50% were considered positive, values between 50% and 60% were regarded as doubtful, and values higher than 60% as negative (thresholds indicated by dashed lines).

At day 14 after infection, the animals were euthanized and sera tested for the presence of VP7-specific antibodies using a commercially available competitive ELISA (Figure 5). All animals seroconverted following challenge infection irrespective of the vaccine group tested, indicating that even in protected animals (groups VSV*ΔG(VP2) and VSV*ΔG(VP2,VP5)) limited replication of challenge virus must have occurred, which was sufficient to trigger an immune response against VP7. In contrast, all immunized sheep were tested negative for VP7 antibodies prior to infection, indicating that the VRP vaccine fully complied with the DIVA principle.

## Discussion

The BTV outer capsid proteins VP2 and VP5 provide potential targets for neutralizing antibodies with all neutralizing epitopes recognized to date residing on VP2. The present study has demonstrated that recombinant VSV replicon particles expressing the VP2 protein of BTV-8 induce the production of serotype-specific neutralizing antibodies that protect sheep from disease and viremia following challenge infection with BTV-8. Our data confirm that immunization with VP2 alone is sufficient to induce a protective immune response in vaccinated animals [14,33,34]. VP5 has been reported to enhance the protective immune response if present in the vaccine along with VP2 [16,35]. Employing a canarypox virus vector expressing VP2 and VP5 of BTV-17, protection in sheep was achieved, but the relative contributions of VP2 and VP5 to protection have not been addressed [36]. In a recent study, expression of both VP2 and VP5 by a recombinant equine herpesvirus vector was necessary to protect mice against BTV-8 infection, whereas VP2 alone was not fully protective [37]. In our study, VRP co-expressing VP2 and VP5 did not induce higher neutralizing antibody titers than VRP expressing VP2. However, one animal in the VSV*ΔG(VP2) group did not develop neutralizing antibodies and was not protected, whereas all animals in the VSV*ΔG(VP2,VP5) group remained completely healthy following challenge with BTV-8. The question whether this higher protection rate is attributable to the co-expression of VP2 and VP5 should be addressed by additional experiments with a larger number of animals. A critical parameter for the efficacy of vector vaccines could be the level of expression and conformation of the recombinant VP2 antigen. If expression levels are low, the proportion of properly folded VP2 might increase in the presence of VP5 given their extensive conformational interaction [13]. However, if high expression levels of VP2 are achieved with a given vector system, the amounts of correctly folded VP2 might be sufficient to induce neutralizing antibodies. Antibodies directed to VP5 may not have neutralizing properties on their own, probably because this antigen is not readily accessible for antibodies on the virion surface. Accordingly, the VP5 primary sequence is much more conserved than that of VP2, indicating that VP5 is subject to less immune pressure.

Conventional BTV vaccines have to be properly inactivated and formulated with adjuvants before they can be applied to animals once (sheep) or twice (cattle). Neutralizing antibodies were shown to persist up to four years after immunization [38,39]. The present VRP vaccine did not depend on adjuvants to induce a protective immune response in sheep, probably because this RNA virus vector is cytotoxic and provides sufficient innate signaling to stimulate the immune system. Any adverse side effects which could be caused by adjuvants [40] are thereby eliminated. However, significant levels of neutralizing serum antibodies were detected only after the animals had been vaccinated with the VRP vaccine twice. Additional studies are required to determine the protective effect of a single immunization. In addition, it will be interesting to see for how long VRP-induced neutralizing antibodies will persist in sheep.

Despite their efficacy, live-attenuated BTV vaccines have inherent disadvantages including transmission to insect vectors, lack of attenuation, reassortment of gene segments with field strains of the virus, and potential to cross the placenta to cause reproductive losses and teratogenic effects [41]. In contrast, the propagation-defective VRP vaccine does not cause disease nor will it be able to revert to virulence. As the G protein, the major antigen of VSV, is not expressed by VRPs, neutralizing antibodies against the viral vector are not induced. Accordingly, VRP vaccines are effective when used repeatedly [25,26,42]. Since antibodies directed to VSV G protein are lacking, vaccinated animals will appear seronegative in VSV diagnostic tests [43].

Vector vaccines are superior to conventional vaccines, since they can serve as marker vaccines [22]. The VRP vaccine did not contain the VP7 antigen and therefore allowed the discrimination of infected from vaccinated animals using a commercially available VP7 ELISA. In contrast, conventional (inactivated) BTV vaccines, live-attenuated and propagation-defective BTV vaccines [44,45] do not comply with the DIVA principle. This has complicated the serological surveillance of BTV in those European countries that implemented a vaccination campaign to control the BTV-8 outbreak in 2006. Moreover, the inability to discriminate between infected and vaccinated animals had a significant impact on economy due to restrictions on animal trade [46]. The recombinant replicon particles eliminate this problem as they can be used as marker vaccines.

Currently, there is no universal vaccine available that would protect against all 26 known serotypes of BTV. The VRP technology is not restricted to BTV-8 antigens but may be used to express VP2 antigen of other BTV serotypes as well. Thus, in case a new serotype emerges in a non-endemic region, a recombinant VRP vaccine matching this serotype could be rapidly produced and used for emergency vaccination. This should be particularly valuable for BTV strains for which inactivated vaccines are not readily available because the corresponding viruses do not propagate well in cell culture. Furthermore, this generic vaccine platform may be also employed to protect horses against infection with African horse sickness virus, an orbivirus of which 9 different serotypes are known.

In summary, this study has demonstrated that propagation-incompetent VSV replicon particles can efficiently protect a natural host against bluetongue disease and viremia. This safe and adjuvant-free vaccine technology complies with the DIVA principle, can be easily adapted to other serotypes and viruses, and is rapidly available in case of an emerging BTV outbreak.

## Competing interests

The authors declare that they have no competing interests.

## Authors’ contributions

SK performed practical and writing work throughout the study in partial fulfillment of the requirements for the D.V.M.-Ph.D. degree from the University of Berne (Graduate School for Cellular and Biomedical Sciences). NR participated in serum neutralization assays, RT-qPCR and ELISA. MH contributed to study design, data analysis and manuscript editing. GZ conceived the study, participated in its design and coordination and helped to draft the manuscript. All authors read and approved the final manuscript.
